# A Stepped Frequency Sweeping Method for Nonlinearity Measurement of Microresonators

**DOI:** 10.3390/s16101700

**Published:** 2016-10-13

**Authors:** Yumiao Wei, Yonggui Dong, Xianxiang Huang, Zhili Zhang

**Affiliations:** 1 State Key Laboratory of Precision Measurement Technology and Instruments, Department of Precision Instrument, Tsinghua University, Beijing 100084, China; weiym13@mails.tsinghua.edu.cn; 2 High-Tech Institute of Xi’an, Xi’an 710025, China; zhangyang8195@126.com (X.H.); effortyao@163.com (Z.Z.)

**Keywords:** MEMS resonators, nonlinear features, Hilbert transform, Backbone curve, singular spectrum analysis

## Abstract

In order to measure the nonlinear features of micromechanical resonators, a free damped oscillation method based on stair-stepped frequency sinusoidal pulse excitation is investigated. In the vicinity of the resonant frequency, a frequency stepping sinusoidal pulse sequence is employed as the excitation signal. A set of free vibration response signals, containing different degrees of nonlinear dynamical characteristics, are obtained. The amplitude-frequency curves of the resonator are acquired from the forced vibration signals. Together with a singular spectrum analysis algorithm, the instantaneous amplitudes and instantaneous frequencies are extracted by a Hilbert transform from the free vibration signals. The calculated Backbone curves, and frequency response function (FRF) curves are distinct and can be used to characterize the nonlinear dynamics of the resonator. Taking a Duffing system as an example, numerical simulations are carried out for free vibration response signals in cases of different signal-to-noise ratios (SNRs). The results show that this method displays better anti-noise performance than FREEVIB. A vibrating ring microgyroscope is experimentally tested. The obtained Backbone and FRF curves agree with those obtained by the traditional frequency sweeping method. As a test technique, the proposed method can also be used to for experimentally testing the dynamic characteristics of other types of micromechanical resonators.

## 1. Introduction

Micromechanical resonators have been widely used as the key sensing elements in diverse sensors such as micromechanical inertial sensors, resonant pressure sensors, etc. Microresonators, fabricated by bulk silicon process and bonding technology, have the advantages of small size, light weight, low power consumption, and suitability for integration with IC technologies. Compared with conventional macroscopic-sized resonators, micromechanical resonators are more liable to present nonlinear dynamic features [1,2]. Since the resonators are small in size, they are generally driven close to or even into nonlinear regimes in order to achieve higher vibration amplitudes and therefore sufficiently higher sensitivity and signal-to-noise ratios (SNRs). However, when the vibration amplitude is close to or even located in the nonlinear region, instability caused by nonlinear effects will emerge and the overall sensor performance will be decreased [3,4]. Therefore, studies on the nonlinear dynamics of microresonators have been a research hot spot in recent years [5,6,7,8]. Obtaining the nonlinear dynamics by experimental methods will not only benefit in operating circuit configuration for the manufactured microsensors, but also provide guidance for future resonator design optimization. Furthermore, experimentally obtained nonlinear dynamic parameters provide reliable support for realizing the parameter resonance of microresonators employing the nonlinear characteristics [5,6,7,8].

With respect to the nonlinear dynamics test for a second order mass-spring-damping system, there are mainly two kinds of experimental approaches, i.e., the sine wave frequency sweeping method and the free damped oscillation method. The former is the most commonly used. The nonlinear features are characterized by analyzing the amplitude-frequency response curves obtained from different excitation amplitudes [9]. Due to the requirement for multiple frequency sweeping operations, the method is practically complicated and cumbersome. The analysis results also strongly rely on the physical model. In contrast, the free damped oscillation method is nonparametrically operated and independent of the physical model. The resonator under test is excited by sinusoidal pulses with a certain duration time and the nonlinear information is obtained from the free vibration signals [10,11]. Within the relevant studies on free damped oscillation methods, FREEVIB proposed by Feldman is the best known algorithm [11,12,13]. Instantaneous parameters, including instantaneous frequency and instantaneous amplitude, are extracted from the free vibration signal by a Hilbert transform. Then the Backbone curve, damping curve, and other nonlinear characteristics of the system are figured out. The main advantages of FREEVIB lay in its extensive applicability and no need for prior knowledge. However, since the Hilbert transform is very sensitive to noise, many references are mainly focused on theoretical analyses and numerical simulations [12,13]. Peng et al. drew on the ideas of Feldman and obtained the nonlinear characteristics from free vibration signals with a Polynomial Chirplet Transform. The numerical simulation results give a preferable anti-noise performance [14,15]. Wang et al. accurately obtained the instantaneous frequency from time-varying free vibration signals by a wavelet ridge method, but the instantaneous amplitude calculation results were not given [16]. To the best of our knowledge, the investigated objects of such Hilbert transform algorithms are mainly macro-sized mechanical structures that are generally operating at low frequencies and with small quality factors. There are fewer relevant reports about microresonators such as MEMS gyroscopes or MEMS accelerometers, etc., whose operating frequencies and quality factors are both relatively high.

In this paper a stepped frequency sweeping method is investigated, focusing on measuring the nonlinearity of microresonators with high frequency and low damping ratio. In the vicinity of the resonant frequency, a frequency stepping sinusoidal pulse sequence is used as the excitation signal. A set of response signals, which contain different levels of nonlinear dynamic characteristics, are therefore obtained experimentally. The amplitude-frequency curves are obtained from the forced vibration signals. From the free vibration signals, the instantaneous frequency (IF) and instantaneous amplitude (IA) are calculated by a Hilbert transform. A singular spectrum analysis (SSA) algorithm is employed to remove the noisy fluctuations from the calculated IFs and IAs. Concentrated and legible Backbone curves and FRF curves can therefore be obtained. Taking the vibrating ring microgyroscope as an example, the nonlinear dynamics of the micromechanical resonator are experimentally analyzed. Satisfactory consistency between the results obtained by the proposed method and those obtained by the traditional frequency sweeping method is obtained.

## 2. Nonlinear SDOF System Response under Stepped Frequency Sweeping Excitation

Under a sinusoidal excitation with finite duration time, the dynamic equation of a single degree-of-freedom (SDOF) second-order vibration system can be expressed as:

(1) $$m\ddot{x}+c(\dot{x})\dot{x}+k(x)x=F(t),\;F(t)=\left\{ \begin{array}{l} B\sin(2\pi f_{d}t),t\leq t_{1};\\ 0,t>t_{1}; \end{array}\right.$$

where *m*, $c\left(\dot{x}\right)$, k(x) denote the mass, damping coefficient and stiffness coefficient, respectively. *F*(*t*) is the excitation signal. *B*, $f_{d}$ are the amplitude and frequency of the sinusoidal excitation signal, respectively. *t*_1_ is the duration time of the sinusoidal signal. For *t > t*_1_, the excitation is stopped and the system comes into the free damped oscillation state.

**Figure 1:**
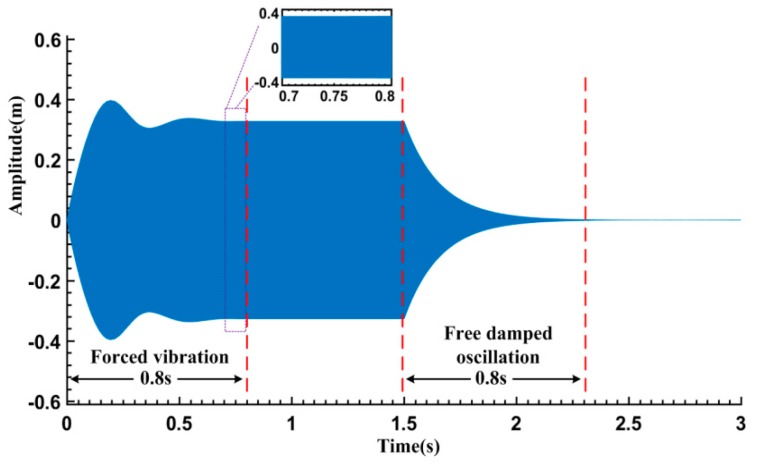
Typical response of the simulated Duffing system.

A high frequency and low damping Duffing system is taken as an example, where *m* is 10^−7^ kg, and $c\left(\dot{x}\right)$ is 1.257 × 10^−6^ N/(m/s) independent of the vibration amplitude. k(x) is vibration amplitude-dependent and equals to 1.579 × 10^3^ × (1 − 0.002*x*^2^) N/m to give a “softened-spring” nonlinearity. *B*, $f_{d}$, *t*_1_ are set to be 0.1 N, 20 kHz and 1.5 s respectively. The sampling frequency $f_{s}$ and the simulation time are set to be 1 MHz and 3 s, respectively. With the aid of the ode45 solver in Matlab, the time series of the system response is numerically calculated as shown in Figure 1. Evidently, in the forced vibration stage, the signal amplitude gradually turns into a relatively steady state after a transition of regular increase and fluctuation. As the amplitude fluctuation rate during 0.7–0.8 s is less than 0.5%, it is appropriate to assume that the vibration enters into the steady-state after that. Additionally, after the excitation is ceased for about 0.8 s, the free damped oscillation amplitude is reduced from 0.33 m to 2.16 × 10^−3^ m. Therefore, the duration time of both the excitation and the sampled free damped oscillation are set to be 0.8 s in the following analysis, unless otherwise indicated.

**Figure 2:**
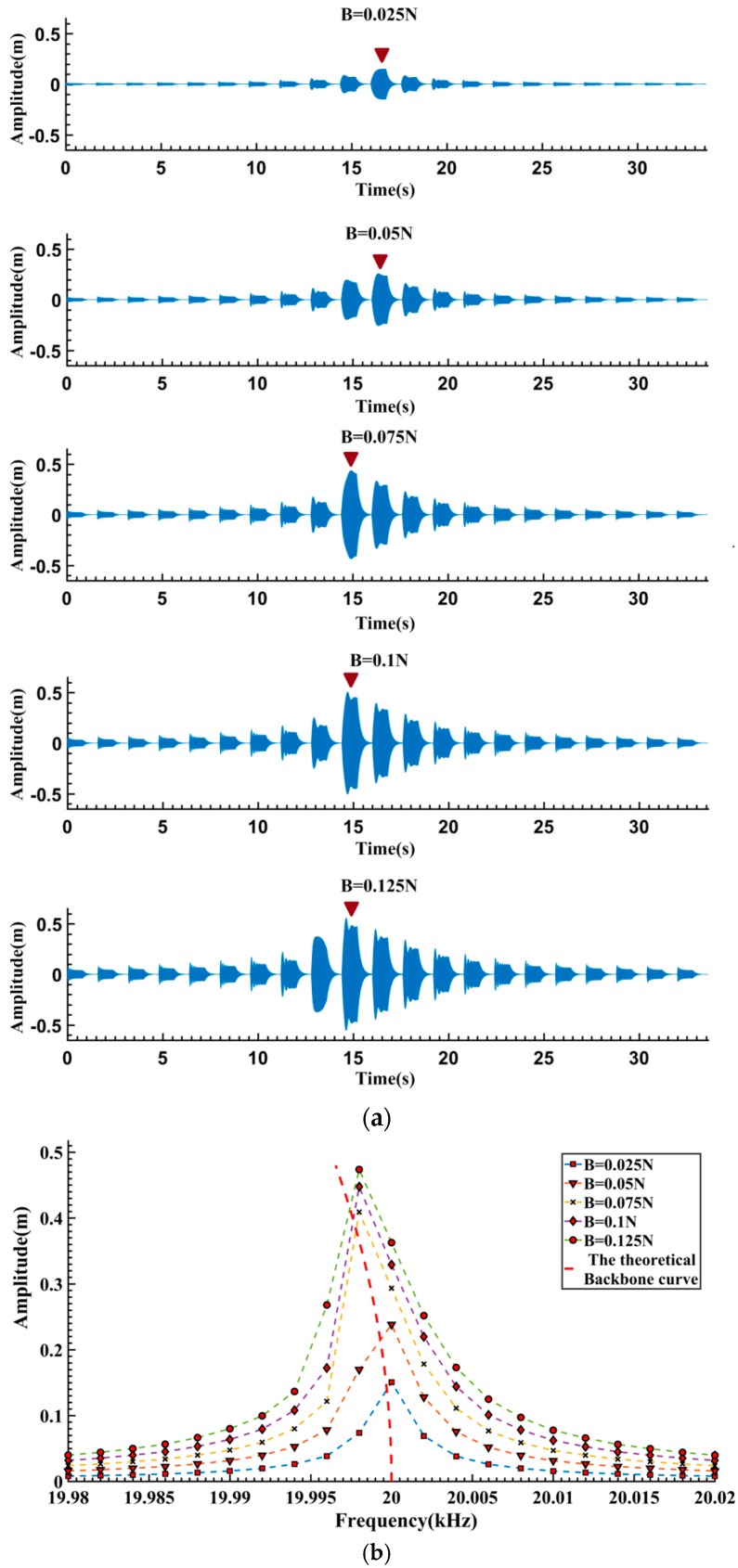
System response signals and amplitude-frequency curves corresponding to different excitation: (**a**) System response signals; (**b**) Amplitude-frequency curves.

The MEMS resonator of an inertial sensor typically works in a low pressure encapsulation setup. Its resonant frequency is generally on the order of tens of kHz and the quality factor is relatively large (Q > 10,000). Therefore, if the excitation frequency is distant from its resonant frequency or the excitation amplitude is not high enough, it is difficult to obtain sufficient vibration amplitude. In order to obtain response signals containing different levels of nonlinear dynamical characteristics, a set of stair-stepped frequency sinusoidal pulse sequences is used as the excitation signal. The excitation amplitude *B* is set to be 0.025–0.125 N, and the excitation frequency $f_{d}$ varies with a 2 Hz step size in the range of 19.980–20.020 kHz. The simulated response signals are illustrated in Figure 2a. It can be seen that even under the same excitation amplitude, there are noticeable differences between the steady-state vibration amplitudes. The highest amplitude appears at the position where the excitation frequency is near the resonant frequency (marked as 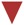 in Figure 2a), while relatively lower amplitudes are observed in other positions. When the system is excited at lower amplitude as shown in Figure 2a (*B* = 0.025 N, 0.05 N), the response amplitude shows a bilaterally symmetrical shape, indicating that system is operating in an approximately linear region. On the other hand, the resonant frequencies (marked with 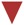 in Figure 2a) shift leftward and bilateral asymmetrical shapes appeared under larger excitation amplitudes (*B* ≥ 0.075 N). It can be therefore concluded that softening nonlinearity has appeared in the system.

From Figure 2a, the average amplitude during 0.7–0.8 s in the forced vibration stages is calculated as the steady-state response amplitude. The obtained relationship between the excitation frequency and steady-state response amplitude is shown in Figure 2b. As a comparison, the theoretical Backbone curve of the system is plotted in the figure as well. Obviously, the obtained amplitude-frequency curves fit the theoretical Backbone curve well.

**Figure 3:**
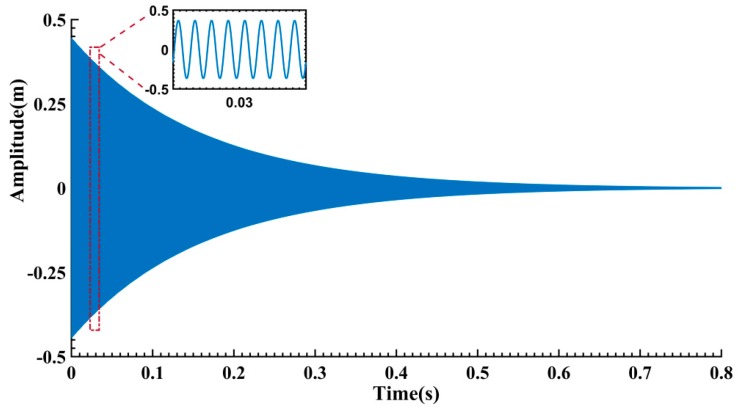
Typical free vibration signal.

The free vibration signal *x*(*t*) is truncated from the largest response segment (marked by 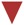 in Figure 2a, B = 0.075 N) as shown in Figure 3. To obtain the nonlinear characteristics from *x*(*t*), the procedure of FREEVIB algorithm [11,12,13] is summarized as follows:

Step 1: We apply the Hilbert transform to *x*(*t*), and $\tilde{x}\left(t\right)$ is obtained by the formula $\tilde{x}\left(t\right)=H\left[x\left(t\right)\right]=\frac{1}{\pi }\int_{-\infty }^{\infty }\frac{x\left(\tau \right)}{t-\tau }d\tau$. $\tilde{x}\left(t\right)$ can be considered as a filter that shifts each frequency component of *x*(*t*) by −π/2 in phases. The analytic signal is constructed in the form of $X\left(t\right)=x\left(t\right)+j\tilde{x}\left(t\right)$. Then, the instantaneous amplitude *A*(*t*) and instantaneous angular frequency *ω*(*t*) are computed by Equation (2):
  (2) A(t)=x2(t)+x˜2(t); ω(t)=x(t)x˜˙(t)−x˙(t)x˜(t)A2(t)=Im[X˙(t)X(t)];
Step 2: Calculate the instantaneous frequency *f*_0_(*t*) and normalized damping coefficient *h*_0_(*t*) from the instantaneous amplitude *A*(*t*), *ω*(*t*) and their derivatives.
  (3) $$f_{0}(t)=\frac{1}{2\pi }\sqrt{\omega^{2}(t)-\frac{\ddot{A}(t)}{A(t)}+\frac{2\dot{A}{\left(t\right)}^{2}}{A{\left(t\right)}^{2}}+\frac{\dot{A}(t)\dot{\omega }(t)}{A(t)\omega (t)}};\;h_{0}(t)=-\frac{\dot{A}(t)}{A(t)}-\frac{\dot{\omega }(t)}{2\omega (t)};$$
Step 3: Smoothing process for *f*_0_(*t*), *A*(*t*) and *h*_0_(*t*) is implemented by low-pass filters and the results are denoted as *f*_0_(*t*)*_LPF_*, *A*(*t*)*_LPF_* and *h*_0_(*t*)*_LPF_*, respectively. Taking *f*_0_(*t*)*_LPF_* as horizontal axis and *A*(*t*)*_LPF_* as vertical axis, the Backbone curve is obtained. The Frequency Response Function (FRF) is estimated at the same time.

**Figure 4:**
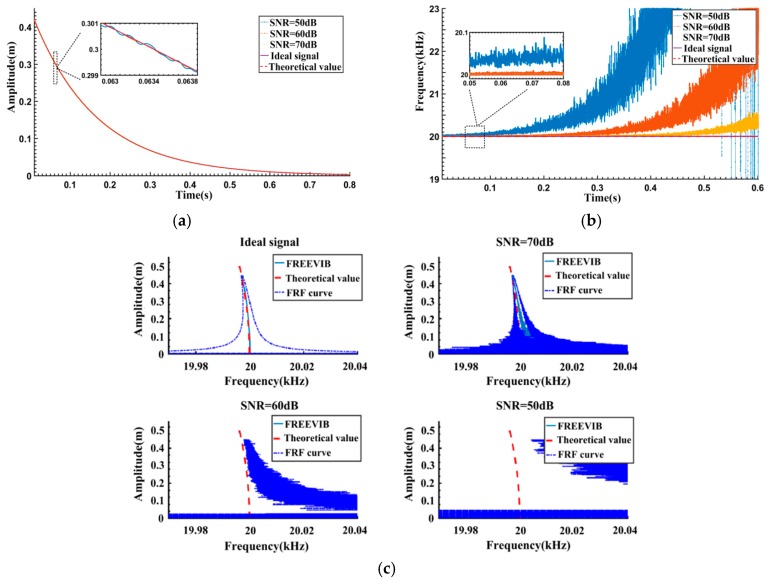
Results corresponding to different SNRs obtained by the FREEVIB method: (**a**) Instantaneous amplitudes (IAs); (**b**) Instantaneous frequencies (IFs); (**c**) Backbone and FRF curves.

In order to simulate practical measurements, white noise is added into the free vibration signal to obtain noisy signals with different SNRs. The results calculated by FREEVIB are given in Figure 4. It can be seen from Figure 4a,b that, along with the decrease of SNR, the variation of the IAs is almost negligible. However, the IFs fluctuate severely and gradually deviate from the theoretical value. Correspondingly, as can be seen from Figure 4c, FREEVIB could accurately extract the Backbone curve from the ideal signal, and the calculated FRF curve is both regular and legible. When the SNR is 70 dB, the upper part of the Backbone curve agrees well with the theoretical value. However, there is a certain offset at the bottom of the Backbone curve and a remarkable fluctuation of the FRF curve. As SNR is reduced to 50 dB, an overall and significant deviation is observed in the results. The FREEVIB algorithm fails to describe the actual dynamic behavior of the system.

Based on the analysis of Figure 4, it could be concluded that the severe fluctuations of *f_0_*(*t*) account for the deviation of Backbone and FRF curves. The low-pass filter, employed in FREEVIB algorithm and based on frequency filtering principle, should be the main cause of the remarkable fluctuation in the results.

In order to further suppress the fluctuations of IFs and extract the frequency change trend effectively, the singular spectrum analysis (SSA) [17,18] algorithm is adopted here. Firstly, the trajectory matrix (Hankle matrix) is constructed from the time series of the IF, *IF*(*n*) = {*p*_1_, *p*_2_,…, *p_N_*}:

(4) $$Y=\left[Y_{1},Y_{2},\ldots ,Y_{K}\right]={\left(y_{ij}\right)}_{i,j=1}^{L,K}\;=\left(\begin{array}{cccc} p_{1} & p_{2} & \cdots & p_{K}\\ p_{2} & p_{3} & \cdots & p_{K+1}\\ \vdots & \vdots & \ddots & \vdots \\ p_{L} & p_{L+1} & \cdots & p_{N} \end{array}\right)$$

where *L* is the window length and *K = N – L +* 1. Then the trajectory matrix is reconstructed by the first *q* principal components after singular value decomposition (SVD): $\hat{Y}=\sum_{i=1}^{q}\sqrt{\lambda_{i}}u_{i}v_{i}$. Finally the smoothing IF is obtained by performing diagonal averaging on $\hat{Y}$ and the reconstruction process. Combining with SSA, the procedure of FREEVIB algorithm is modified as follows:

Step 1: Calculate the instantaneous amplitude *A*(*t*) and the instantaneous phase *θ*(*t*) from the free vibration signal *x*(*t*) by the Hilbert transform:
  (5) $$A(t)=\sqrt{x^{2}(t)+{\tilde{x}}^{2}(t)};\;\theta (t)=\arctan(\hat{x}(t)/x(t));$$
Step 2: Figure out the instantaneous frequency *f*(*t*) after the differential operation on *θ*(*t*), and the expression is *f*(*t*) = 0.5*π*^−1^*dθ*(*t*)/*dt*. The estimation of IA and IF, indicated by *A*(*t*)*_SSA_* and *f*(*t*)*_SSA_* respectively, are obtained by processing *A*(*t*) and *f*(*t*) with SSA algorithm.
Step 3: Compute the normalized damping coefficient *h*(*t*) by *A*(*t*)*_SSA_*, *f*(*t*)*_SSA_* and their derivatives [19]. *f_estd_*(*t*) is figured out by the above mentioned parameters, and the FRF curve is further obtained. The corresponding equations are as follows:
  (6) $$\begin{array}{l} h(t)=-\frac{A{\dot{(}t)}_{SSA}}{A{\left(t\right)}_{SSA}}-\frac{f{\dot{(}t)}_{SSA}}{2f{\left(t\right)}_{SSA}};\\ f_{estd}^{2}(t)=f{\left(t\right)}^{2}{}_{SSA}-\frac{2h{\left(t\right)}^{2}}{4\pi^{2}}\pm \frac{f{\left(t\right)}_{SSA}h(t)}{\pi }\sqrt{\frac{\max(\dot{A}{\left(t\right)}^{2}{}_{SSA})}{A{\left(t\right)}^{2}{}_{SSA}}+\frac{h{\left(t\right)}^{2}}{4\pi^{2}f{\left(t\right)}^{2}{}_{SSA}}-1}; \end{array}$$

**Figure 5:**
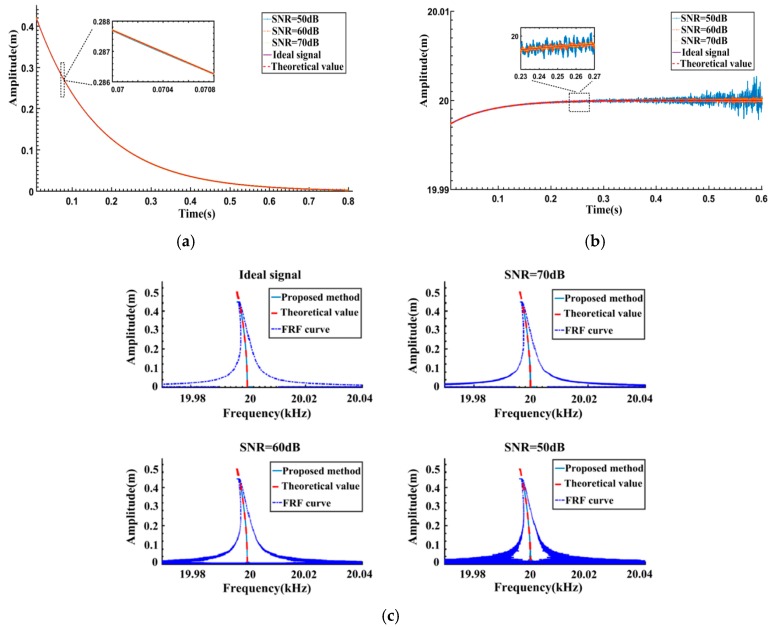
Results corresponding to different SNRs obtained by the proposed method: (**a**) Instantaneous amplitudes (IAs); (**b**) Instantaneous frequencies (IFs); (**c**) Backbone and FRF curves.

The same noisy free vibration signals as in Figure 4 are processed and the results are given in Figure 5. The window length is set to be 800 sample points in the SSA algorithm. For the ideal signal, the Backbone curve and FRF curve are both extracted accurately by the proposed method. Significant improvements are obtained for the noisy signals case. Unlike the FREEVIB algorithm, when the SNR becomes relatively low, there are no longer noticeable fluctuations in IFs. Consequently, the calculated Backbone curves remain legible and accordant with the theoretical values. As the SNR decreases, there are some fluctuations in the computed FRFs, but it is still possible to effectively represent the system dynamics.

**Figure 6:**
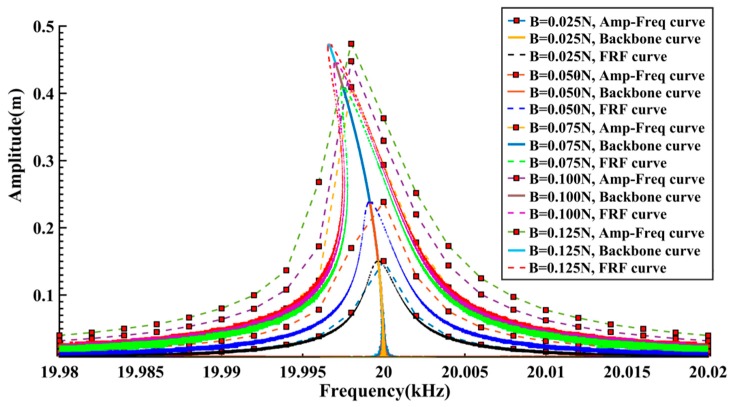
Comparison of the simulation results.

Further calculations are carried out with the other free vibration signals marked 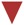 in Figure 2a. After truncation and noise addition, five segments of free vibration signals with 60 dB SNR are processed by the proposed algorithm and the results are given in Figure 6. It can be seen that, in the case of lower excitation amplitude (B = 0.025 N, 0.05 N), almost no visible nonlinearity appears both in the Backbone curves and FRF curves, indicating that the system is operating in the approximately linear region. With the increase of excitation amplitude (B = 0.075 N–0.125 N), the nonlinear features in the Backbone curves and FRF curves become significant. Moreover, the variation tendencies of both the Backbone curves and FRF curves indicate the “softened-spring” characteristics of the system.

In order to perform a comparison, the Backbone curves and FRF curves under different excitation conditions are also summarized in Figure 6. The amplitude-frequency curves in Figure 2 are given as well. It can be seen that the calculated Backbone curves and FRF curves show good consistency. The amplitude-frequency curves, on the other hand, differ slightly from the FRF curves. This difference may be caused by the relatively larger frequency intervals as well as the fact only approximate steady-state data are used for the calculations. In any case, the shapes of the amplitude-frequency curves are still in good agreement with the FRF curves.

## 3. Experiments and Discussion

**Figure 7:**
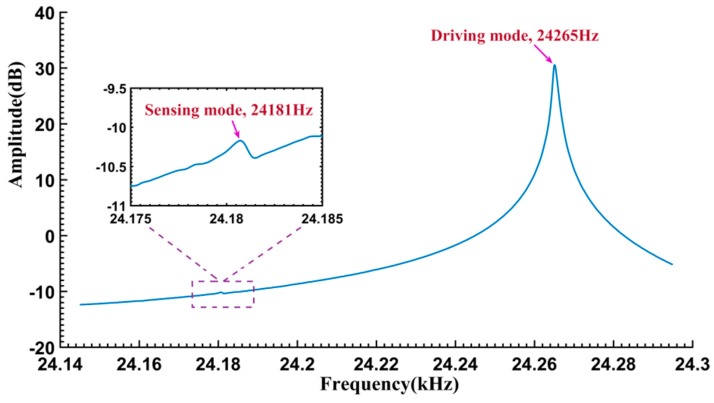
Amplitude-frequency response of the driving mode.

A MEMS vibrating ring gyroscope developed by our laboratory [20] was experimentally tested. Its resonator structure and working principle are similar to those reported in [21,22,23,24,25]. The resonator is a suspended ring that works in its fundamental flexural mode. In operation, the ring is electrostatically driven and its vibration is capacitively picked up by the sensing electrodes (45° from the driving electrodes). The resonator is encapsulated in a low-pressure metallic package, and the Q factor is approximately 20,000. Due to the imperfections in the bulk silicon manufacturing process, there is an obvious frequency split of about 100 Hz in the practically fabricated gyroscopes. For the one under test, the amplitude-frequency response curve of the driving mode is presented in Figure 7. Its natural frequency of the sensing mode is 24,181 Hz, but that of the driving mode is 24,265 Hz. In our case, the resonator is tested near the resonant frequency of the driving axis, so the influence of the sensing mode is quite weak and negligible, and the free vibration can be considered as a mono-frequency oscillation of a SDOF system.

**Figure 8:**
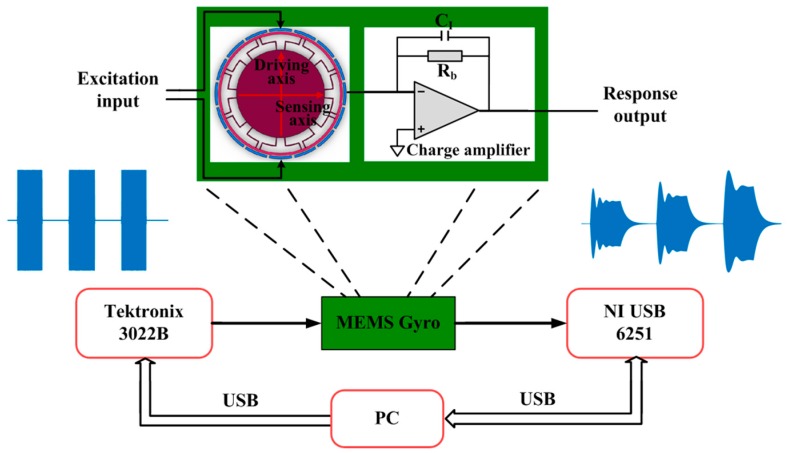
Experimental testing system.

The schematic diagram of the testing system is shown in Figure 8. The output terminal of an AFG 3022B function generator (Tektronix, Beaverton, OR, USA) is connected to the driving electrodes. The vibration along the driving axis is driven by the generated excitation signals. The vibration along the sensing axis is picked up by the sensing electrodes and the charge amplifier to give the response output signal. Therefore, the vibration response amplitude is given in voltages instead of meters. A USB 6251 DAQ device (NI, Austin, TX, USA) is used to sample both the excitation and response signals synchronously. The sampled signals are transmitted to a computer through an USB interface for subsequent signal processing and analysis.

During experiments, a stair-stepped frequency sinusoidal pulse sequence is generated by the AFG 3022B and used as the excitation signal. The excitation frequency $f_{d}$ varies in a 0.5 Hz forward step within the range of 24.257–24.269 Hz. The amplitudes of the excitation signals are varied from 50 mV to 200 mV. Similar to the above mentioned simulations, the time durations of the forced and free damped oscillation are set to be 1.6 and 1.8 s, respectively. The sampling frequency $f_{s}$ is set to be 250 kHz (approximately 10 times the signal frequency).

**Figure 9:**
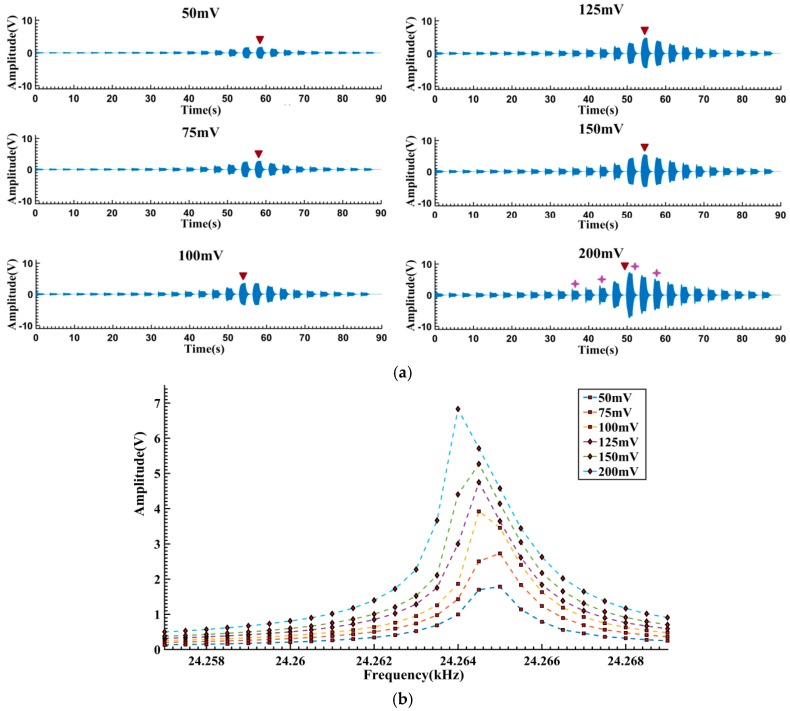
Response of the tested gyroscope under different excitation voltages: (**a**) Measured response signals; (**b**) Amplitude-frequency curves.

The measured response signals and the calculated amplitude-frequency curves are given in Figure 9. Like the simulation results, the largest response amplitudes appear at positions where the excitation frequency is near the resonant frequencies (marked by 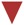 in Figure 9a), and relatively smaller amplitudes are observed in other positions. When the excitation voltage exceeds 100 mV, the resonant frequencies shift leftwards and bilateral asymmetrical shapes appear, revealing the “softened-spring” characteristics of the tested gyroscope.

**Figure 10:**
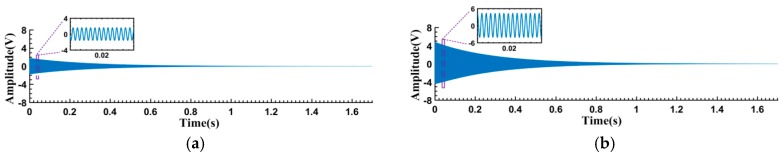

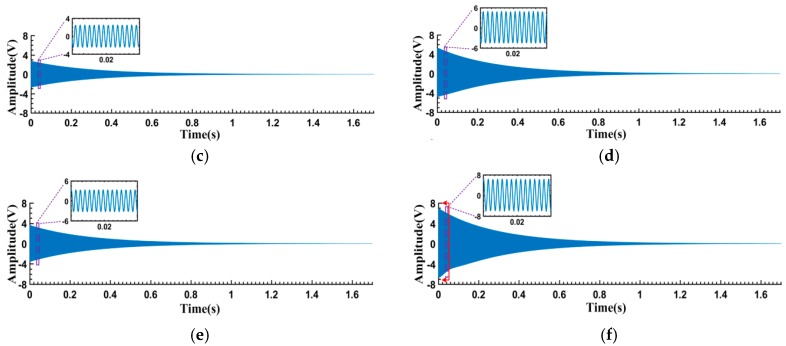
Free vibration signals of the largest response segments in Figure 9: (**a**) 50 mV; (**b**) 75 mV; (**c**) 100 mV; (**d**) 125 mV; (**e**) 150 mV; (**f**) 200 mV.

The free vibration signals of the largest response segments (marked as 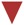 in Figure 9a) are truncated as shown in Figure 10. It can be seen that when the vibration amplitudes are relatively small (Figure 10a–e), the signal waveforms are vertically symmetrical and exponential decayed. However, when the vibration amplitudes exceed a certain level (about 6 V, indicated by the left arrow in Figure 10f), the asymmetry gradually becomes visible. This might be caused by the non-ideal structure of the micromachined resonator. Asymmetry of the fabricated structure leads to different dynamics characteristics when the resonator vibrates positively and negatively from the balance position. Therefore, the asymmetric data on the left side of the indicating arrow in Figure 10f are excluded to avoid the latent impact of waveform asymmetry.

**Figure 11:**
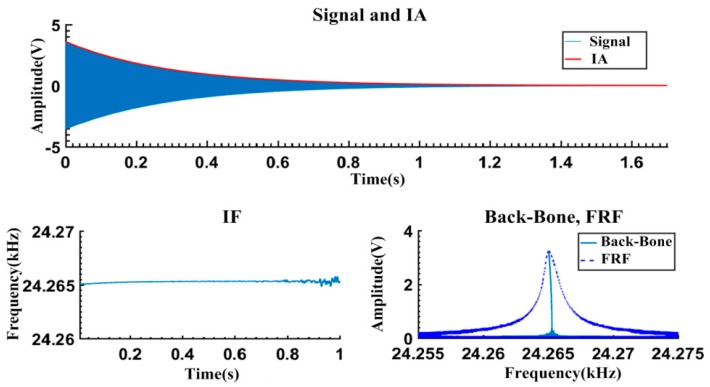
Calculated results for response signals of Figure 10c.

**Figure 12:**
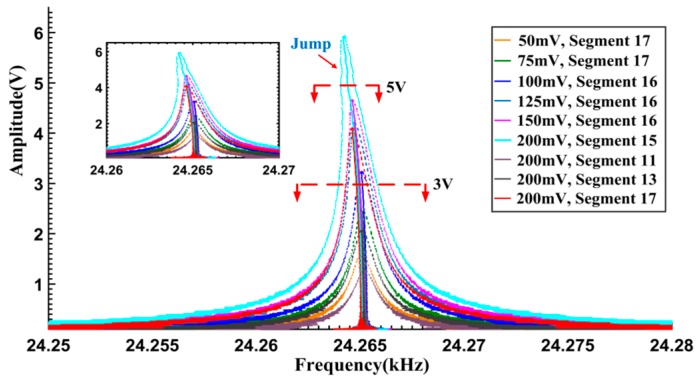
Backbone curves cluster and FRF curves cluster.

The free vibration signals in Figure 10 are processed by the proposed method. The results are shown in Figure 11. As an example, Figure 11 gives the detailed results of Figure 10c. Both the calculated IA and IF curves are satisfactorily smooth. Correspondingly, the calculated Backbone curves and FRF curves are concentrated and legible, and can be used for characterizing the dynamic features. Six sets of the extracted Backbone and FRF curves are given in Figure 12. When the maximum response amplitudes are below 3 V, the Backbone curves are approximate plumb lines and the FRF curves are bilaterally symmetrical, indicating that the resonator is vibrating linearly. As the maximum amplitudes increase to 4–6 V, the Backbone curves gradually bend to the left. In addition, “softened-spring” features are also exhibited in the FRF curves.

In order to further verify the results, the response signals under 200 mV excitation in Figure 9a are analyzed. Four typical segments of the free vibration signals (segments 11, 13, 15, 17, marked as 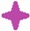 in Figure 9a) are truncated and processed. The obtained Backbone curves and FRF curves are given in Figure 12. Approximately linear performances can be seen in the curves of segments 11, 13, while those of segments 15, 17 reveal the softened nonlinearity. In practical terms, if the excitation amplitude is properly selected, the linearity and non-linearity of the system dynamics can be obtained in a single measurement cycle.

It is obvious that the processed results in Figure 12 agree with each other. In summary, when the maximum response amplitudes are less than 3 V, the Backbone curves appear as plumb lines, indicating that the resonator is working in the linear region. On the other hand, when the maximum response amplitudes exceed 3 V, the calculated curves start to bend leftward, representing the “softened-spring” nonlinearity of the tested gyroscope. When the maximum amplitudes reach about 6 V, it can be seen from the FRF curve, there will be multivalued mapping relationships between the excitation frequencies and the response amplitudes in the left vicinity of the resonant frequency. If the gyroscope is working in that region, it is liable to induce the occurrence of frequency instability. The gyroscope stability will therefore be greatly decreased in such a case. Taking the equilibrium between SNR improvement and nonlinearity effects into account, 3–6 V is considered to be an ideal region for operating the circuit setup. This conclusion is confirmed by the practical circuit adjustment results performed in our laboratory. Optimized performance, especially regarding the stability, is obtained when the output amplitude of the gyroscope is set to be 4.5–5 V.

**Figure 13:**
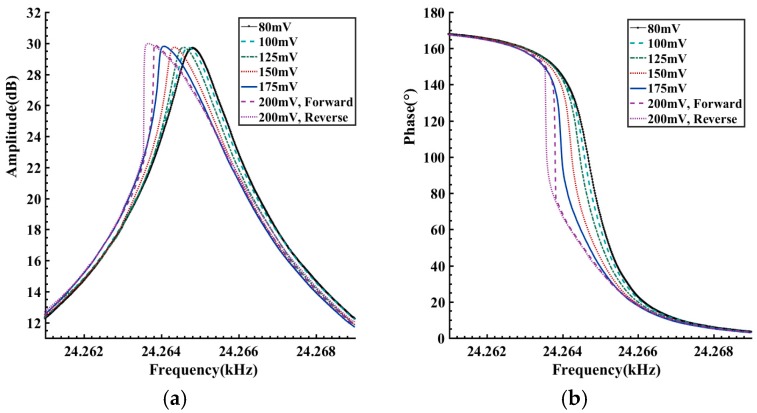
Frequency response curves obtained by frequency sweeping: (**a**) Amplitude-frequency curves; (**b**) Phase-frequency curves.

In order to verify the abovementioned results, a frequency sweeping method is employed to acquire the dynamic characteristics of the tested gyroscope. A HP35670A dynamic signal analyzer (Agilent, Santa Clara, CA, USA) is used in the experiments. The excitation voltages are assigned to six values in the 80–200 mV range. Within the range of 24,257–24,269 Hz, a forward and reverse frequency sweeping operation is conducted. The frequency step and the holding time are set to be 0.01 Hz and 1.5 s. The measured frequency response curves are given in Figure 13.

**Figure 14:**
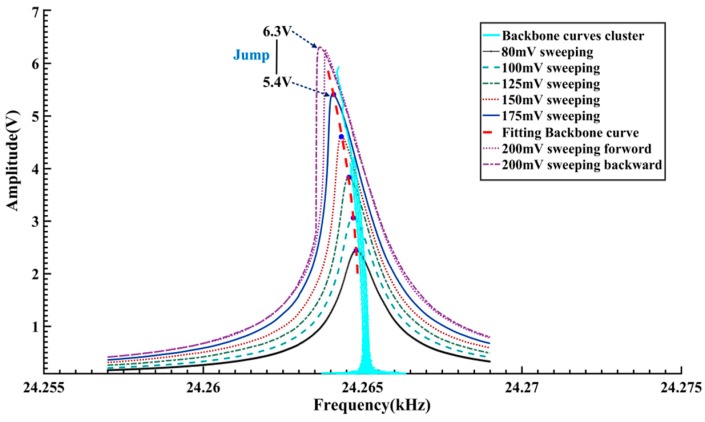
Comparison between the Backbone curves cluster and frequency sweeping measurement results.

**Figure 15:**
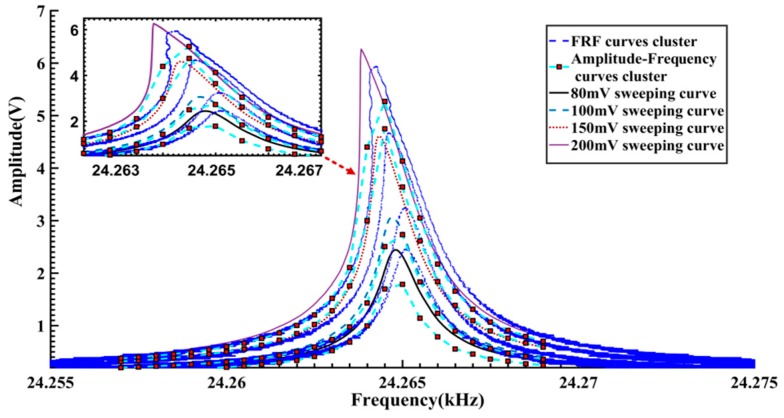
Contrast between FRF curves cluster, Amplitude-frequency curves cluster and frequency sweeping results.

When the amplitude of the excitation voltage is within 80–175 mV, there is little difference between the forward and reverse sweeping curves. However, as the excitation amplitude reaches up to 200 mV, because of the frequency-jumping phenomenon, a significant difference is observed between the forward and reverse sweeping curves. The voltage-frequency curves, as shown in Figure 14, are obtained by conversion of the amplitude-frequency responses in Figure 13a. As the vibration amplitude increases, there is an apparent tendency of leftwards bending in the voltage-frequency curves, reflecting the softening nonlinearity of the gyroscope. When the vibration voltage is about 5.4–6.3 V, the frequency jumping phenomenon occurs. These results are quite consistent with the analyses of Figure 12. As a comparison, the Backbone curves cluster in Figure 12 is also plotted in Figure 14. It can be seen that on the whole the Backbone curves obtained by the proposed method agree with those obtained by the frequency sweeping experiments. Compared with the frequency sweeping results, overall the obtained Backbone curves are biased towards the right by about 0.2 Hz. Figure 15 gives another comparison of the FRF curves cluster, amplitude-frequency curves cluster in Figure 9b and the frequency sweeping results. It is quite clear that there is a good consistency in the trend and shape of the resulting curves. Like the Backbone curves in Figure 14, compared with the other two kinds of processed curves, the calculated FRF curves show a slight rightwards deviation. Considering the distinction of the measurement conditions, such a difference should be acceptable. Specifically speaking, during the frequency sweeping operation, the gyroscope under test works in a steady excitation and response way.

Besides the mechanical properties of the resonator, several other electrical factors within the driven circuit will be reflected in the measurement results. On the other hand, only free vibration signals are considered in the proposed method and therefore any influences of such electrical factors will not appear in the results. In this sense, the proposed method is more reliable to characterize the dynamic features of the resonator.

## 4. Conclusions

Aiming at characterizing the nonlinearity of microresonators, a stepped frequency sweeping method is proposed. Near the resonant frequency regime, a frequency stepping sinusoidal pulse sequence is used as the excitation signal. The amplitude-frequency responses are obtained from the forced vibration signals. A set of free vibration signals are acquired, containing different degrees of nonlinear dynamical characteristics. Instantaneous parameters are figured out from the free vibration signals by the Hilbert Transform. The noisy fluctuations of IFs and IAs are removed by a SSA algorithm. The resulting Backbone curves and FRF curves are legible and capable of characterizing the dynamic features.

The simulation results show that the proposed method has better anti-noise performance than FREEVIB. Furthermore, a vibrating ring gyroscope is tested experimentally. The extracted Backbone and FRF curves are in good agreement with those obtained by traditional frequency sweeping method. The processed results can be used as a guidance for operating circuit configuration and design optimization, etc. Particularly, the proposed method, using free damped oscillation signals, can better represent the dynamics features of the resonator than the frequency sweeping method, due to exclusion of other factors. The main advantage of the method lies in its improved convenience and efficiency, i.e., a single measurement is sufficient to obtain results equivalent to multiple frequency sweeping measurements. In addition, the proposed technique, as a practical and economic test method, can also be employed to measure the dynamic characteristics of other micromechanical systems.

## References

[1] Tatar E., Mukherjee T., Fedder G.K. Nonlinearity tuning and its effects on the performance of a MEMS gyroscope. Proceedings of the 18th International Conference on Solid-State Sensors, Actuators and Microsystems (TRANSDUCERS).

[2] Villanueva L.G., Karabalin R.B., Matheny M.H., Chi D., Sader J.E., Roukes M.L. (2013). Nonlinearity in nanomechanical cantilevers. Phys. Rev. B.

[3] Mestrom R.M.C., Fey R.H.B., Phan K.L., Nijmeijer H. (2010). Simulations and experiments of hardening and softening resonances in a clamped–clamped beam MEMS resonator. Sens. Actuators A.

[4] Mestrom R.M.C., Fey R.H.B., Van Beek J.T.M., Phan K.L., Nijmeijer H. (2008). Modelling the dynamics of a MEMS resonator: simulations and experiments. Sens. Actuators A.

[5] Nitzan S.H., Zega V., Li M., Ahn C.H., Corigliano A., Kenny T.W., Horsley D.A. (2015). Self-induced parametric amplification arising from nonlinear elastic coupling in a micromechanical resonating disk gyroscope. Sci. Rep..

[6] Hamed Y.S., El-Sayed A.T., El-Zahar E.R. (2016). On controlling the vibrations and energy transfer in MEMS gyroscope system with simultaneous resonance. Nonlinear Dyn..

[7] Ruzziconi L., Ramini A.H., Younis M.I., Lenci S. (2014). Theoretical prediction of experimental jump and pull-in dynamics in a MEMS sensor. Sensors.

[8] Wang C.C., Yau H.T. (2011). Nonlinear dynamic analysis and sliding mode control for a gyroscope system. Nonlinear Dyn..

[9] Zhiyong C., Bin Z., Rong Z. (2010). Nonlinear vibration of micromachined angular vibration resonator in low pressure. Chin. J. Sci. Instrum..

[10] Sracic M.W., Allen M.S., Sumali H. (2012). Identifying the modal properties of nonlinear structures using measured free response time histories from a scanning laser Doppler vibrometer. Topics in Nonlinear Dynamics.

[11] Feldman M. (2012). Nonparametric identification of asymmetric nonlinear vibration systems with the Hilbert transform. J. Sound Vib..

[12] Feldman M. (2014). Hilbert transform methods for nonparametric identification of nonlinear time varying vibration systems. Mech. Syst. Signal Process..

[13] Feldman M. (2011). Hilbert Transform Applications in Mechanical Vibration.

[14] Yang Y., Peng Z., Dong X., Zhang W., Meng G. (2016). Nonlinear time-varying vibration system identification using parametric time–frequency transform with spline kernel. Nonlinear Dyn..

[15] Peng Z., Meng G., Chu F., Lang Z., Zhang W., Yang Y. (2011). Polynomial chirplet transform with application to instantaneous frequency estimation. IEEE Trans. Instrum. Meas..

[16] Wang C., Ren W.X., Wang Z.C., Zhu H.P. (2013). Instantaneous frequency identification of time-varying structures by continuous wavelet transform. Eng. Struct..

[17] Golyandina N., Zhigljavsky A. (2013). Chapter 2 Basic SSA. Singular Spectrum Analysis for Time Series.

[18] Alexandrov T. A Method of Trend Extraction Using Singular Spectrum Analysis. https://www.ine.pt/revstat/pdf/rs090101.pdf.

[19] Feldman M., Bucher I., Rotberg J. (2009). Experimental identification of nonlinearities under free and forced vibration using the hilbert transform. J. Vib. Control.

[20] Li H. (2015). Research on Muti-Ring Structure Micromachined Gyroscopes. Ph.D. Thesis.

[21] Yoon S.W., Lee S., Najafi K. (2011). Vibration sensitivity analysis of MEMS vibratory ring gyroscopes. Sens. Actuators A.

[22] Challoner A.D., Howard H.G., Liu J.Y. Boeing disc resonator gyroscope. Proceedings of the Position, Location and Navigation Symposium-PLANS 2014.

[23] Zhou X., Li Q., Xiao D., Hou Z., Chen Z., Wu Y., Wu X. The mechanical sensitivity optimization of a disk resonator gyroscope with mutative ring thickness. Proceedings of the 2016 IEEE International Symposium on Inertial Sensors and Systems.

[24] Flader I.B., Ahn C.H., Gerrard D.D., Ng E.J., Yang Y., Hong V.A., Pavone M., Kenny T.W. Autonomous calibration of MEMS disk resonating gyroscope for improved sensor performance. Proceedings of the 2016 IEEE American Control Conference (ACC).

[25] Tao Y., Wu X., Xiao D., Wu Y., Cui H., Xi X., Zhu B. (2011). Design, analysis and experiment of a novel ring vibratory gyroscope. Sens. Actuators A.

